# *Staphylococcal* Protein A Is a Key Factor in Neutrophil Extracellular Traps Formation

**DOI:** 10.3389/fimmu.2018.00165

**Published:** 2018-02-05

**Authors:** Tamara Hoppenbrouwers, Andi R. Sultan, Tsion E. Abraham, Nicole A. Lemmens-den Toom, Silvie Hansenová Maňásková, Wiggert A. van Cappellen, Adriaan B. Houtsmuller, Willem J. B. van Wamel, Moniek P. M. de Maat, Johan W. van Neck

**Affiliations:** ^1^Department of Plastic and Reconstructive Surgery, Erasmus MC, Rotterdam, Netherlands; ^2^Department of Hematology, Erasmus MC, Rotterdam, Netherlands; ^3^Department of Medical Microbiology and Infectious Diseases, Erasmus MC, Rotterdam, Netherlands; ^4^Erasmus Optical Imaging Center, Department of Pathology, Erasmus MC, Rotterdam, Netherlands

**Keywords:** neutrophil extracellular traps, *NETs*, S. aureus, *Staphylococcus aureus*, Protein A, SpA

## Abstract

*Staphylococcus aureus* are strong inducers of neutrophil extracellular traps (NETs), a defense mechanism of neutrophils against pathogens. Our aim was to explore the role of Protein A in *S. aureus*-induced NETosis. We determined the Protein A production of four different *S. aureus* strains and found a direct relationship between the degree of NETosis induction and Protein A production: strains producing higher concentrations of Protein A evoke significantly more NETs. A *S. aureus* strain in which Protein A as well as a second binding protein for immunoglobulins (*Sbi*) have been knocked-out (Δ*SpA* Δ*Sbi*) induced significantly less NETosis than the wild-type strain. NETosis induction by this knockout strain can be rescued by the addition of purified Protein A. Dead *S. aureus* did not induce NETosis. In conclusion, Protein A is a determinant for NETosis induction by *S. aureus*.

## Introduction

Upon encountering bacteria, neutrophils can form neutrophil extracellular traps (NETs) as part of their antimicrobial defense mechanism. During NETosis, neutrophils excrete their DNA into the extracellular space, along with histones and other antimicrobial factors. These NETs trap bacteria and thereby limit bacterial spreading (1, 2). NETs have been shown to play an important role in contributing to several pathological conditions, such as chronic wounds (3), thrombosis (4–6), and sepsis (7–9).

The bacterial inducing capacity of NETosis is different between bacterial species (10). A very potent inducer of NETosis is *Staphylococcus aureus* (11, 12). *S. aureus* is a Gram-positive bacterium that can cause many different infections and, particularly when dealing with methicillin-resistant *S. aureus*, can cause critical problems in hospitals. *S. aureus* possesses multiple evasion strategies against the human immune system, such as the production of immune-modulators (13–17) and the secretion of nucleases, which enables them to escape NETs (16).

*Staphylococcus aureus* also can evade phagocytosing neutrophils by blocking neutrophil rolling on activated endothelial cells and by targeting both antibodies and opsonins, necessary for pathogen recognition by neutrophils (18). One of the main bacterial proteins involved in phagocytosis evasion is Protein A. *Staphylococcal* Protein A (*SpA*) is a 42-kDa large protein which is covalently linked to the *staphylococcal* surface and can be secreted into the extra-bacterial environment (17, 19, 20).

*Staphylococcal* Protein A is known to be able to manipulate or to avoid early host adaptive immune responses. It can bind to the Fcγ domain of IgG and, therefore, inhibit opsonization that precedes phagocytosis (17, 19–21). Furthermore, it can induce apoptosis in B-cells by binding to the Fab regions of the B-cell receptor and act as a B-cell superartigen (22). However, little is known about its direct effect on innate immune cells, particularly neutrophils. Since neutrophils are one of the earliest effector host immune cell against *S. aureus* invasion and because of their ability to form NETs, we were interested to study whether Protein A is also involved in NETosis. To achieve this, we determined the Protein A production in different *S. aureus* strains and its relationship with NETosis inducing capacity. Next, we obtained more insight in the role of Protein A in NETosis by studying the rescue of NETosis with Protein A in a *S. aureus* Protein A knockout strain.

## Materials and Methods

### Bacterial Strains

Bacterial strains used in this study are listed in Table 1. Strains were obtained from the bacterial collection of Department of Medical Microbiology and Infectious Diseases, Erasmus MC Rotterdam.

**Table 1:** Overview of the *Staphylococcus aureus* strains used in this study.

Strain	Genetic background	Description
Newman	ST8	Wild type, laboratory strain
USA300	ST8	Clinical strain
M116	ST238, ST8	Clinical strain
RN6390	ST8	Laboratory strain, derivative of 8325-4
Newman Δ*SpA* Δ*Sbi*	ST8	Laboratory strain, derivate from Newman strain

### Bacterial Growth Condition

All strains were cultured on Trypticase™ Soy Agar (TSA) (Becton Dickinson, Breda, The Netherlands) with 5% sheep blood overnight at 37°C. Protein A and the second binding protein for immunoglobulins (*Sbi*) double knockout Newman strain (Δ*SpA* Δ*Sbi*) was cultured on TSA containing 5 µg/ml gentamycin and 5 µg/ml tetracycline to maintain its knockout status. After an overnight incubation at 37°C, bacteria were suspended in NaCl 0.9% solution (OD 0.5 at OD_600nm_), and 200 µl was added to a sterile Erlenmeyer flask containing 100 ml Iscove’s Modified Dulbecco’s Medium (IMDM) (Gibco, Bleiswijk, The Netherlands).

The flask was then incubated for 24 h at 37°C at 150 rpm. The next day, based on OD_600nm_ measurements, the individual strains were concentrated to reach a final concentration of 2 × 10^10^ bacteria/ml. Heat killed bacteria were generated by incubating the bacteria at 96°C for 10 min. Bacteria were then harvested and transferred to new IMDM medium. To control for the effectiveness of the heat treatment, the heat-killed bacteria were stained with propidium iodide (PI, diluted 1:400, Sigma Aldrich, Zwijndrecht, The Netherlands) and a sample was cultured to check for growth by plating.

### Secreted Protein A Measurement

The concentrations of released Protein A by *S. aureus* strains were measured using a sandwich ELISA type assay specific for Protein A (Enzo, Bruxelles, Belgium) according to the manufacturer’s protocol. The detection range of the kit was 15.6–1,000 pg Protein A/ml. *S. aureus* strains Newman, USA300, RN6390, M116, and Newman Δ*SpA* Δ*Sbi* were cultured as described above and after overnight culturing, 20 µl of the supernatant was collected, centrifuged at 4,000 *g*, and then filtered. Supernatant from Newman Δ*SpA* Δ*Sbi* bacteria was included as a negative control. The optical density at 450 nm was measured using a Biotek plate reader (Biotek) with Gen5 software and used to calculate the protein A concentration.

### FACS Analysis of Surface Associated Protein A

Four milliliters of IMDM were inoculated with an overnight culture of *S. aureus* (Newman, USA300, RN6390, and M116) to obtain OD_600nm_ of 0.05. The individual cultures were incubated for 24 h at 37°C with continuous shaking at 230 rpm. The OD_600nm_ of the bacteria culture was normalized to 0.300, and the bacteria were washed 3 times with PBS, followed by centrifugation for 5 min at 4,000 *g*. The individual bacterial pellets were suspended in 100 µl PBS. Ten microliters of each bacterial suspension were mixed with either 10 µl of 1:50 dilution of anti-protein A IgY-FITC (FITC-labeled Chicken anti-Protein A), (Gallus Immunotech Inc., Fergus, Canada) or with 10 µl PBS, used as negative control, in an U shape 96-well microplate (Greiner Bio-One, Oberösterreich, Germany). The plate was then incubated at 10°C for 45 min at 800 rpm in the dark. After washing 3 times with 200 µl PBS, bacteria were centrifuged for 5 min at 3,500 *g*. Bacteria were suspended in 50 µl of PBS and their fluorescence (emission 488–522 nm) was quantified with Accuri C6 Flow Cytometer and analyzed using Accuri C6 Software (version 1.0.264.21) (both BD Bioscience, Breda, The Netherlands). Values are expressed in mean fluorescence intensity (MFI).

### Neutrophil Isolation

Neutrophils were isolated as described previously (23), under endotoxin-free conditions. Briefly, medium Lymphoprep™ (Stemcell Technologies) was used to isolate neutrophils from blood derived from healthy donors within the age range of 25–50. Donors did not use any medication (e.g., anti-inflammatory drugs) that could influence study results. Red blood cells were lysed using Erythrolysis buffer (155 mM NH_4_Cl, 10 mM KHCO_3_, 1 mM EDTA, pH 7.4) and then neutrophils were washed two times with HEPES buffer. A final neutrophil concentration of 2 × 10^7^ cells/ml was used. All experiments were approved by the Medical Ethics Committee of the Erasmus MC. Each experiment was performed with neutrophils derived from a different donor.

### NETosis Induction and Imaging

Neutrophils were stained for DNA with Hoechst 34580 (diluted 1:10,000, Life Technologies) and for extracellular DNA with PI (diluted 1:400, Sigma Aldrich) in 500 µl DMEM culture medium (Biowhittaker, Lonza). The cells were allowed to attach to gelatin-coated coverslips at 37°C for at least 1 h.

To induce NETosis, 500 µl 2 × 10^10^ bacteria/ml were added to 500 µl 2 × 10^7^ neutrophils/ml in a Attofluor Cell Chamber (Thermo Fisher Scientific, Bleiswijk, The Netherlands). The chamber was sealed, and the neutrophils were continuously imaged with a confocal microscope (Leica SP5 AOBS) with a 40× magnification and numerical aperture (n.a.) of 1.25. Hoechst and PI were excited by 405 nm (emission BP 450–550 nm) and 561 nm (emission 570–620 nm) lasers, respectively. NETs were visible as PI positive elongated structures and were quantified (see NETs Quantification).

In order to study the effect of Protein A on NETosis induction by *S. aureus* Newman Δ*SpA* Δ*Sbi* strain, 100 µl of either 0.01, 0.1, or 1 mg/ml of purified Protein A (Sigma Aldrich, Zwijndrecht, The Netherlands) was added to the Newman Δ*SpA* Δ*Sbi* strain prior to co-incubation with neutrophils (final concentration range 0.9–90 µg/ml Protein A).

### NETs Quantification

*z*-Stack images were taken from randomly distributed fields of view within the cell chamber every 3–5 min, in a time frame of 5–40 min. Every image had the same *X, Y*, and *Z* dimensions. In each image, NETs were manually traced in every *z*-stack by using ImageJ (Version 1.49, National Institutes of Health, USA). The total volume (cubic micrometer) of NETs was calculated from 10 to 15 images per strain. The average percentage of NETs coverage was then calculated: total volume of NETs/total volume.

### Statistical Analyses

Data are presented as mean ± SEM. A statistical unpaired, two-tailed *t*-test with Statistical Package for the Social Sciences (SPSS, IBM, version 21) was used to analyze differences between groups. Results were considered significant when *p* < 0.05.

## Results

**Figure 1:**
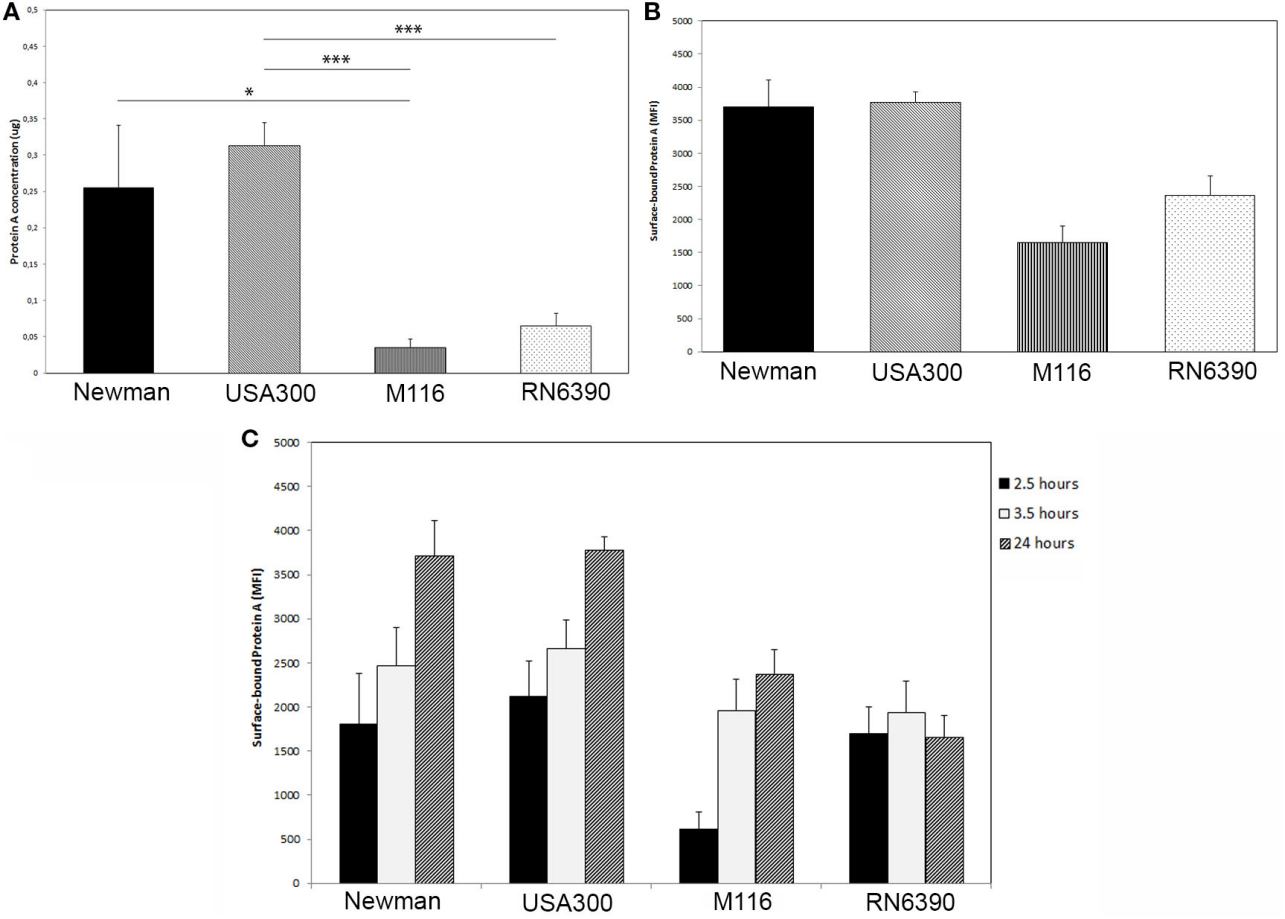
Protein A production by *Staphylococcus aureus*. **(A)** Protein A secretion is higher in *S. aureus* strains Newman and USA300 compared to *S. aureus* strains M116 and RN6390, as determined by an ELISA assay (*n* = 5). **(B)** More Protein A is bound to the surface of *S. aureus* strains Newman and USA300 compared to *S. aureus* strains M116 and RN6390, as determined by FACS (*n* = 3). **(C)** Amount of Protein A bound to the surface of *S. aureus* measured on 2.5, 3.5, and 24 h as determined by FACS. Except for strain RN6390, the amount of surface bound Protein A is increasing over time (*n* = 3, neutrophils derived from three individual donors).

The secretion of Protein A in the supernatant of the overnight grown *S. aureus* USA300 (ST8) was significantly higher than that of M116 and RN6390 (0.31 ± 0.03 µg/ml USA300 vs 0.04 ± 0.01 µg/ml M116, *p* < 0.001 and 0.31 ± 0.03 µg/ml USA300 vs 0.07 ± 0.02 µg/ml RN6390, *p* < 0.001). *S. aureus* Newman secreted significantly more Protein A than M116 (0.26 ± 0.09 µg/ml Newman vs 0.04 ± 0.01 µg/ml M116, *p* = 0.04), and compared to *S. aureus* RN6390, a trend could be observed (0.26 ± 0.09 µg/ml Newman vs 0.07 ± 0.02 µg/ml, *p* = 0.06) (Figure 1A). No Protein A was measured in the Protein A knockout Newman Δ*SpA* Δ*Sbi*.

The amount of Protein A associated to the bacterial surface was higher in Newman and USA300 (3,703 ± 404 MFI and 3,767 ± 163 MFI, respectively) compared to M116 and RN6390 (2,336 ± 288 MFI and 1,649 ± 254 MFI, respectively) (Figure 1B). In literature, it was described that Protein A is in particular expressed in classical bacterial growth media during the exponential growth phase. To determine if this is the case in IMDM, we as well made a growth curve and determined at three time points the amount of surface associated Protein A. Interestingly, the level of the surface associated Protein A during stationary phase (2.5 and 3.5 h) is higher compared to its exponential phase (24 h, Figure 1C), except for strain RN6390.

**Figure 2:**
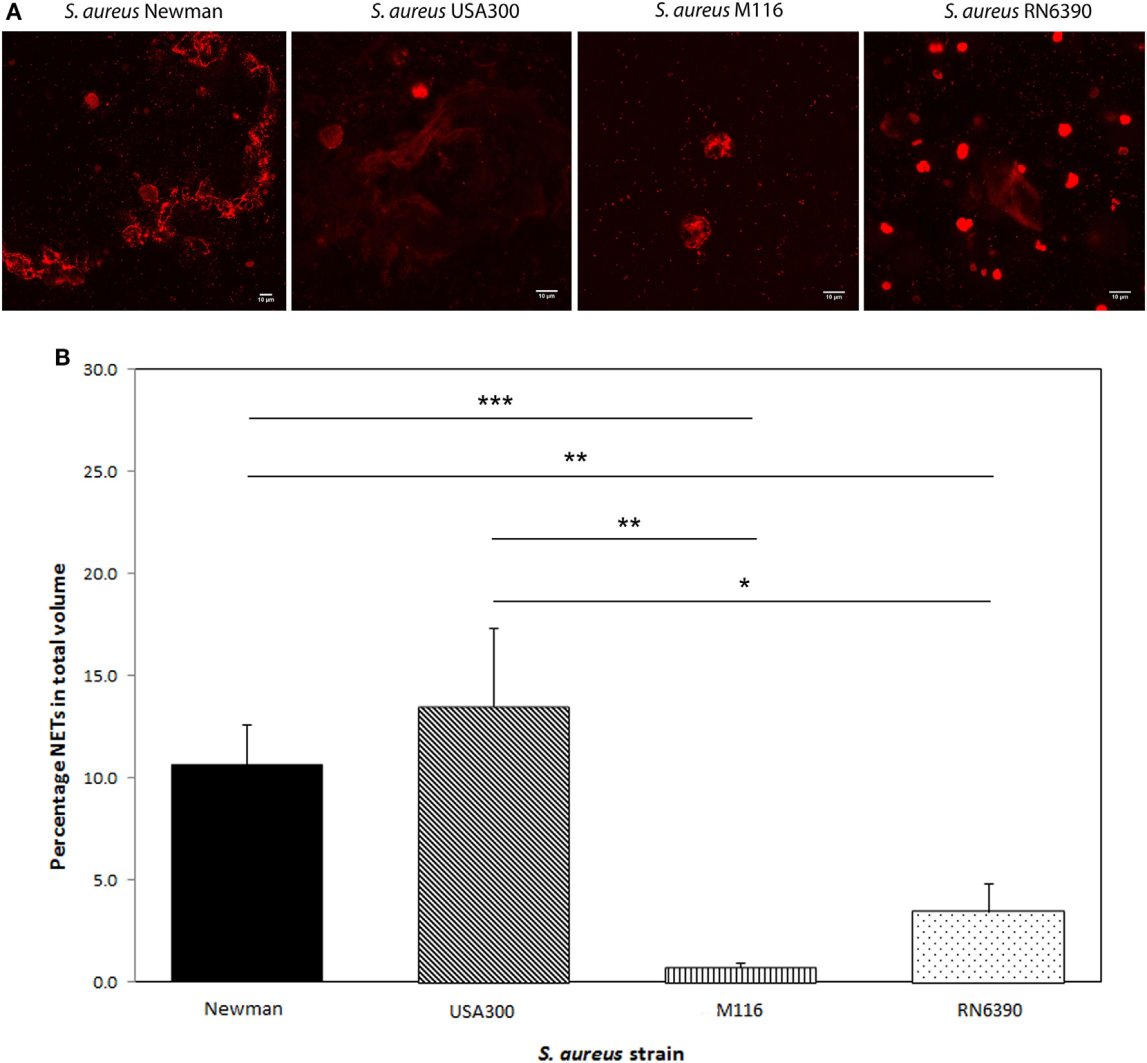
Neutrophil extracellular traps (NETs) induction by different *Staphylococcus aureus* strains Newman, USA300, M116, and RN6390. **(A)** *In vitro* NETs formation as indicated by propidium iodide (red). **(B)** Strains Newman and USA300 induce significantly more NETs than M116 and RN6390, as indicated by percentage of NETs coverage in the total volume. Results of three separate experiments, neutrophils were derived from three individual donors (*p < 0.05; **p < 0.01; ***p < 0.001).

When we tested these *S. aureus* strains to see whether the amount of Protein A plays a role in NETosis, we observed a positive correlation between Protein A levels and NETosis. High Protein A-producing strains Newman and USA300 induced significantly more NETosis (Newman 10.7 ± 1.9% and USA300 13.5 ± 3.8% of the total volume) compared to the low Protein A-producing strains M116 (0.7 ± 0.2%, *p* < 0.001 and *p* = 0.003, respectively) and RN6390 (3.4 ± 1.4%, *p* = 0.005 and *p* = 0.02, respectively) (Figure 2). Corresponding NETs volumes are 3,750 ± 675 µm^3^ (Newman), 4,738 ± 1347 µm^3^ (USA300), 245 ± 72 µm^3^ (M116), and 1,208 ± 482 µm^3^ (RN6390). NETosis in all strains started within 5 min and within 40 min all neutrophils had formed NETs.

**Figure 3:**
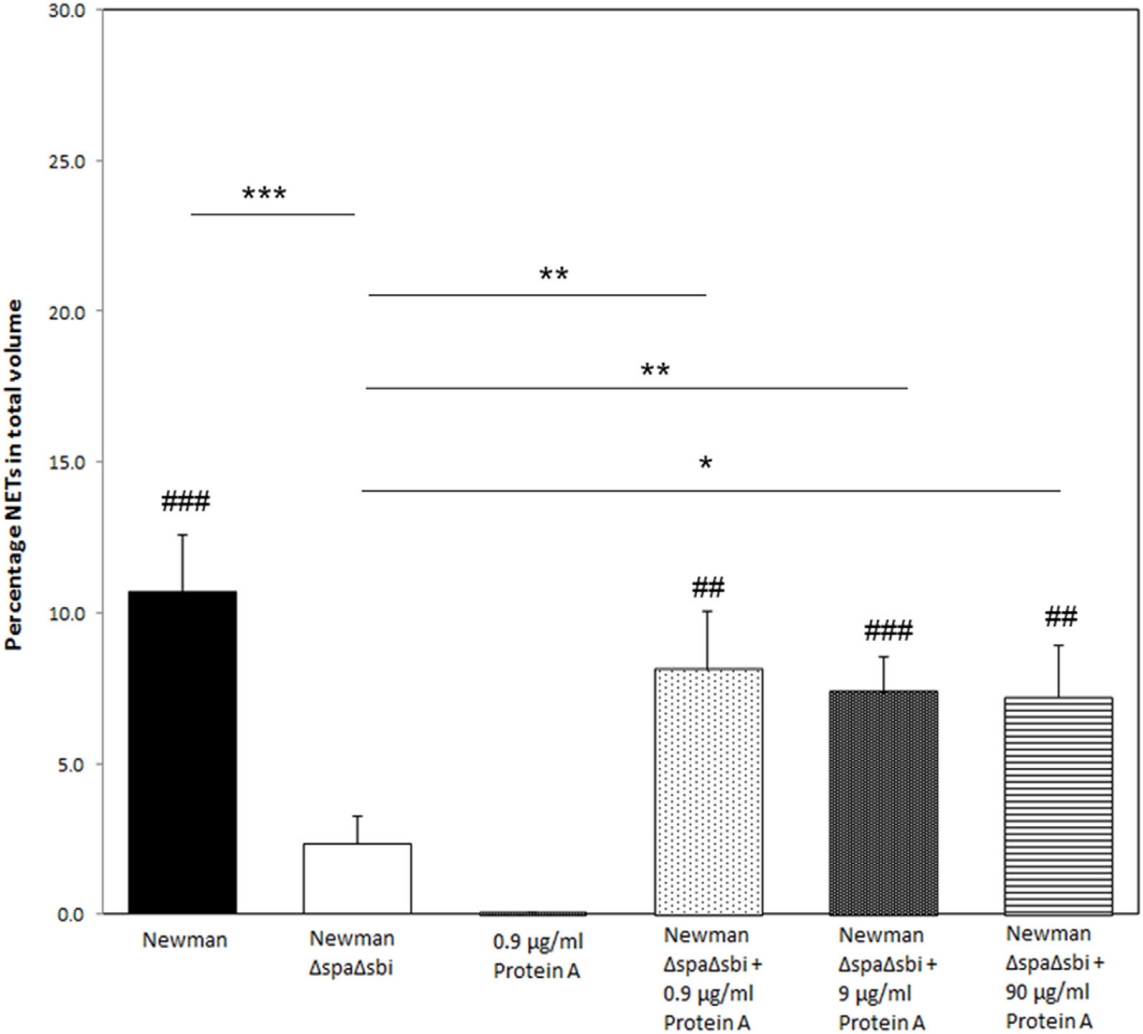
Reversed effect of significantly reduced NETosis by *Staphylococcus aureus* knockout strain after the addition of purified Protein A. Except for *S. aureus* Newman Δ*SpA*Δ*Sbi*, all conditions induce more NETosis than Protein A only. Results of three separate experiments, neutrophils were derived from three individual donors. * indicates significant difference from *S. aureus* Newman Δ*SpA*Δ*Sbi*. ^#^ indicates significant difference when compared to neutrophils stimulated with 0.9 µg/ml Protein A (*/^#^*p* < 0.05; **/^##^*p* < 0.01; ***/^###^*p* < 0.001).

To further determine the contribution of Protein A to NETosis induction, we used a *SpA Sbi* double knockout strain of *S. aureus* Newman (Δ*SpA* Δ*Sbi*) to induce NETosis. Only very modest NETosis was observed (2.3 ± 0.9%), which was significantly lower than of the WT Newman strain (*p* < 0.001). We could recover NETosis by the knockout strain by adding purified Protein A. Addition of 0.9, 9, or 90 µg/ml of purified Protein A to the knockout strain, prior to NETosis induction, rescued NETosis induction (8.1 ± 1.9%, *p* = 0.01; 7.4 ± 1.2%, *p* = 0.005; 7.2 ± 1.7%, *p* = 0.02, respectively) (Figure 3) to comparable levels that were observed for WT Newman strain (*p* = 0.36, *p* = 0.26, and *p* = 0.19, respectively).

**Figure 4:**
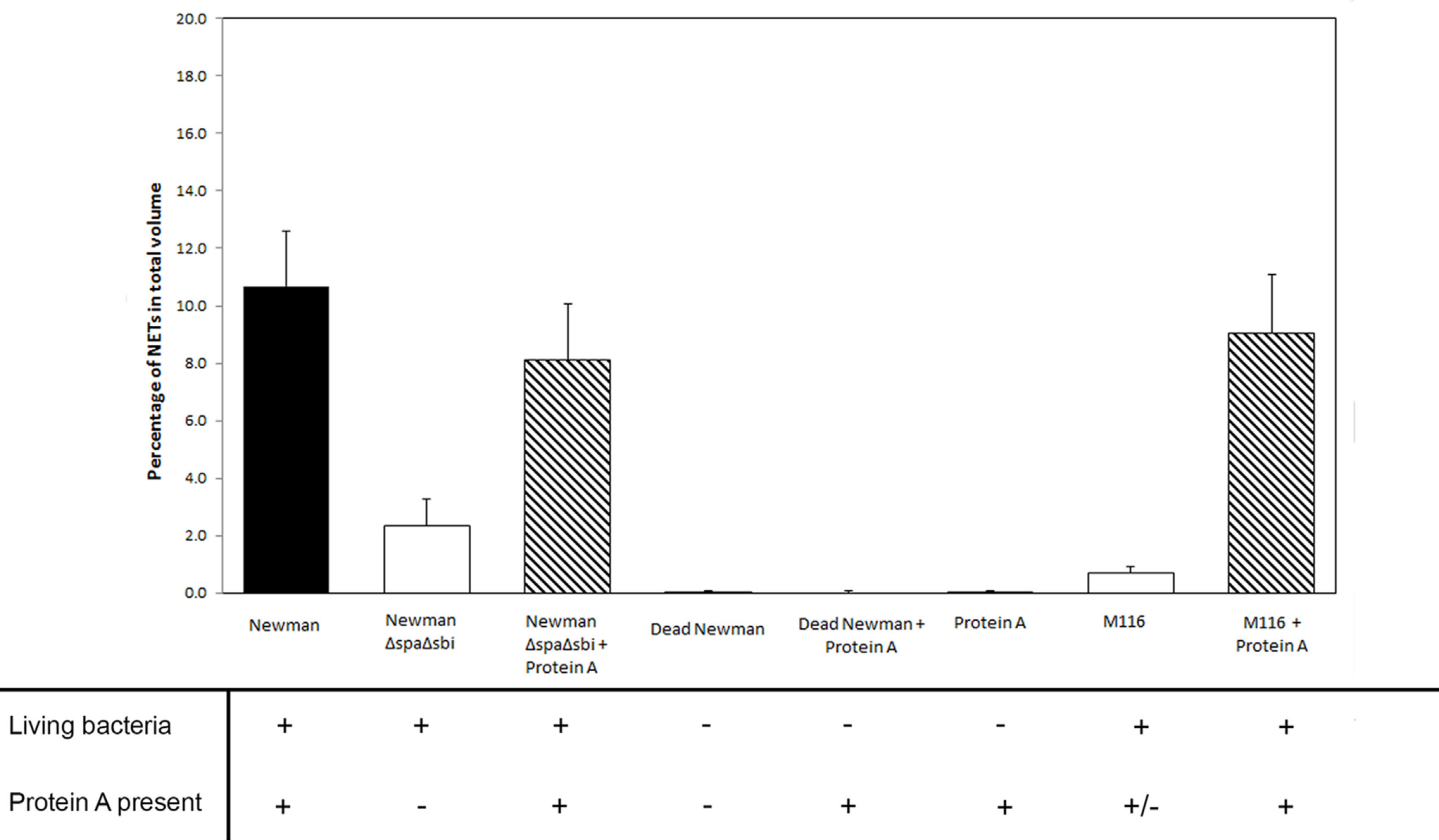
The effect of live and dead *Staphylococcus aureus* and the presence of Protein A (both produced by *S. aureus* and added) on NETosis in different rescue experiments.

Additionally, we also were able to rescue NETosis formation to the level induced by WT Newman when Protein A was added to the modestly NETosis inducing strain M116 (0.7 ± 0.2% in M116 vs 9.1 ± 2.0% in M116 plus Protein A, *p* < 0.001), giving it the same NETosis induction rate compared to WT Newman (*p* = 0.63, Figure 4).

**Figure 5:**
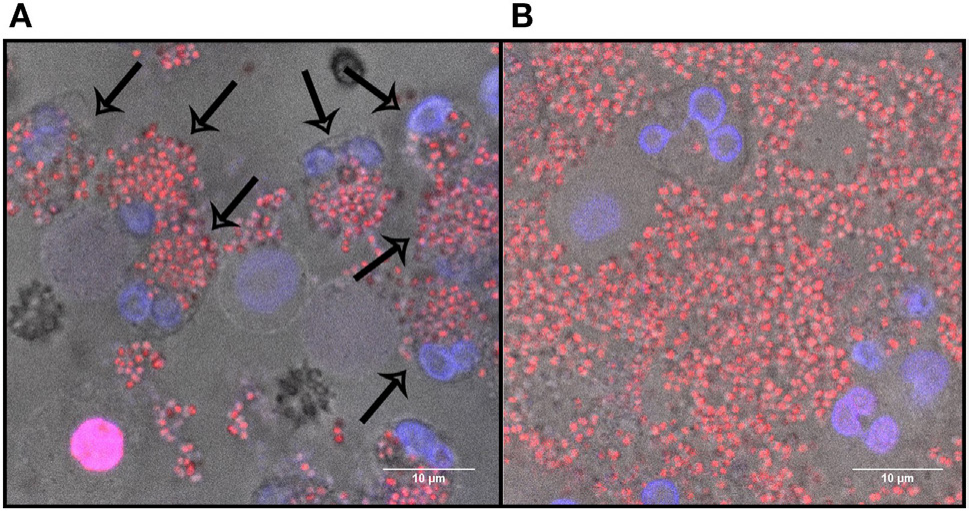
The effect of Protein A on dead bacteria in the presence of neutrophils after 40 min of incubation. **(A)** Dead *Staphylococcus aureus* Newman incubated with neutrophils. Arrows indicate phagocytosis. **(B)** Dead *S. aureus* Newman incubated with 0.9 µg/ml Protein A and neutrophils. Blue, DNA; red, dead bacteria.

No NETosis was observed when 0.9, 9, or 90 µg/ml of purified Protein A was added to the neutrophils without bacteria (0.0 ± 0.0, Figure 3). To explore the effect of bacterial viability on NETosis induction, 0.9 and 9 µg/ml Protein A were added to dead bacteria (WT Newman). No NETosis was observed (Figure 4) which indicates that living bacteria are needed in order to induce NETosis. We observed that dead bacteria were phagocytosed by neutrophils, however, when 0.9–90 µg/ml Protein A was added, bacteria were not cleared by the neutrophils (Figure 5).

## Discussion

In this study, we demonstrated that Protein A secretion is positively correlated to NETosis induction by *S. aureus*. Previously, it has been described that different bacterial species induce different NETosis responses (10, 12, 23). We showed that between different *S. aureus* strains, differences in NETosis response are seen, which is correlated to the amount of Protein A present. Furthermore, NETosis induction by *S. aureus* with little to no Protein A present can be rescued by adding Protein A.

In our study, the bacterial cell wall-associated protein A level is higher during the stationary phase than during the exponential phase. In a previous study by Gao et al., using trypticase soy broth (TSB) medium, the *spa* gene, that encodes Protein A in *S. aureus*, is upregulated in the exponential growth phase of the bacteria and the expression could be regulated *via* various factors (24). In our experiments, the use of IMDM over TSB is preferred, since we work with neutrophils and the composition of culture media for mammalian cells, such as IMDM, is more closely resembling the composition of human conditions. *S. aureus* behaves differently in IMDM than when cultured in TSB medium (25), which is an interesting observation.

Protein A is one of the important virulence factors that *S. aureus* uses to evade the immune system. By Protein A binding to the Fcγ region of immunoglobulin IgG, bacteria can avoid being opsonized and phagocytosed by neutrophils and other immune cells, as it inhibits binding to the neutrophils Fc receptor (26). In previous experiments, we already reported that dead bacteria are phagocytosed by neutrophils and do not induce NETosis (23). Also, in line with previous experiments (23), no spontaneous NETs were formed within 1 h of induction, as we showed that <1% of NETs were formed when either dead bacteria or Protein A alone was added to the neutrophils. We now observed that phagocytosis was inhibited when Protein A was added to dead bacteria, although in our experiment, to our knowledge, no IgG was present. Protein A has also been described to bind to the TNFR1 of immune cells, causing production of pro-inflammatory cytokines, such as IL-6 and TNF-α (27). Both of these cytokines have been described as inducers of NETosis (28, 29). In addition, Protein A binding to TNFR1 can cause inhibition of proliferation, mineralization, apoptosis, and activation in osteoclasts, indicating that also in neutrophils, binding to TNFR may activate NETosis. Also, Protein A can activate NADHP oxidase (30), on which NETosis depends (31). However, in our experiments no NETosis was observed when Protein A was added to the neutrophils without bacteria, indicating that another stimulus, for example a different cofactor secreted by living *S. aureus*, is needed in addition to Protein A.

The observations in our study that Protein A is both a determinant of NETs formation and can inhibit phagocytosis without IgG are interesting additions to previous studies, where living *S. aureus* bacteria were reported to degrade the NETs (16, 32). These studies speculated that the ability of NETosis induction by bacteria might be a function of bacteria to kill human neutrophils, due to the high survival of bacteria that were able to escape the NETs. Our findings contribute to this hypothesis and suggest that *S. aureus* is not only able to evade phagocytosis by secreting Protein A, but also to eliminate the neutrophils by evoking NETosis, from which they can escape by producing nucleases.

In this study, we used the double knockout strain *S. aureus* Newman for SpA and Sbi. The NETosis inducing capacity of this strain also might have been affected by Sbi. Sbi has a similar function and structure as Protein A, and it can interact with complement protein C3, thereby inhibiting opsonization and, therefore, phagocytosis (32, 33). However, in our experiments, we were able to fully rescue NETosis induction by only adding purified Protein A, indicating that Protein A is the important determinant of NETosis in this experimental setting.

In chronic wound infections such as osteomyelitis and ulcers, *S. aureus* is one of the most frequently isolated bacteria (3, 34). Also in other diseases where *S. aureus* infection plays a major role, such as sepsis (7) and COPD (35–38), more NETs have been associated with a higher severity of the disease. By understanding more about direct interaction between the human immune system, in this case neutrophils, and *S. aureus*, we find more ways to efficiently target the bacteria by interfering with the bacterial products that influence and inhibit our immune system. In conclusion, the amount of Protein A present is an important determinant of NETosis induction by *S. aureus*, and NETosis inducing capacities of strains with little to no Protein A present can be enhanced by addition of commercial Protein A.

## Author Contributions

All authors contributed to the experimental design. Experiments were performed by TH, AS, TA, NT, and SM. Data were processed by TH, AS, TA, and WC. All authors contributed to data interpretation. Figures were generated by TH and were interpreted by all authors. Statistical analyses were performed by TH and AS. The manuscript was written by TH and AS and revised by all authors.

## Conflict of Interest Statement

The authors declare that the research was conducted in the absence of any commercial or financial relationships that could be construed as a potential conflict of interest.
